# Particle Swarm Optimization with Double Learning Patterns

**DOI:** 10.1155/2016/6510303

**Published:** 2015-12-27

**Authors:** Yuanxia Shen, Linna Wei, Chuanhua Zeng, Jian Chen

**Affiliations:** School of Computer Science and Technology, Anhui University of Technology, Maanshan 243002, China

## Abstract

Particle Swarm Optimization (PSO) is an effective tool in solving optimization problems. However, PSO usually suffers from the premature convergence due to the quick losing of the swarm diversity. In this paper, we first analyze the motion behavior of the swarm based on the probability characteristic of learning parameters. Then a PSO with double learning patterns (PSO-DLP) is developed, which employs the master swarm and the slave swarm with different learning patterns to achieve a trade-off between the convergence speed and the swarm diversity. The particles in the master swarm and the slave swarm are encouraged to explore search for keeping the swarm diversity and to learn from the global best particle for refining a promising solution, respectively. When the evolutionary states of two swarms interact, an interaction mechanism is enabled. This mechanism can help the slave swarm in jumping out of the local optima and improve the convergence precision of the master swarm. The proposed PSO-DLP is evaluated on 20 benchmark functions, including rotated multimodal and complex shifted problems. The simulation results and statistical analysis show that PSO-DLP obtains a promising performance and outperforms eight PSO variants.

## 1. Introduction

Particle Swarm Optimization (PSO) [1, 2], firstly proposed by Kennedy and Eberhart in 1995, was inspired by the simulation of simplified social behaviors including fish schooling and bird flocking. Similar to genetic algorithm, it is also a population-based algorithm, but it has no evolutionary operations such as crossover, mutation, or selection. PSO finds the global best solution by adjusting the trajectory of each particle not only towards its personal best particle* pbest* but also towards the historically global best particle* gbest* [3]. Recently, PSO has been successfully applied to optimization problems in many fields [4–7].

In the basic PSO [1], each particle in the swarm learns from* pbest* and* gbest*. During the evolutionary process,* gbest *is the only shared information acquired by the whole swarm, which finally leads to all particles converging to the same destination and the diversity losing quickly. If* gbest *is a local optimum far from the global one, the swarm is easy to be trapped in local optimum. The learning mechanism of the basic PSO can cause a fast convergence rate, but it easily leads to the premature convergence when solving multimodal optimization problems. In order to overcome this problem, researchers proposed many strategies to improve it.

An adaptive strategy of the learning parameter [3, 8–18] is an effective way to improve the PSO performance. Shi and Eberhart [8] proposed a linearly decreasing inertia weight (LDIW) to balance the local search and global search. Ratnaweera et al. [3] proposed a time-varying acceleration coefficient (TVAC), which is beneficial to enhancing the exploration ability of particles in the early evolutionary phase and improving the local searching ability of particles in the late phase. In [3], the two variants of the PSO-TVAC were developed, namely, the PSO-TVAC with mutation (MPSO-TVAC) and the self-organizing hierarchical PSO-TVAC (HPSO). Zhan et al. [9] proposed an adaptive PSO in which the learning parameters were adaptively adjusted with the change of the evolutionary states of the swarm. Kundu et al. [10] proposed a nonlinearly time-varying acceleration coefficient and an aging guideline to avoid the premature convergence. They also suggested a mean learning strategy to enhance the exploitation search.

To increase the swarm diversity, auxiliary techniques are introduced into PSO's framework, such as genetic operators [3, 12, 13, 19], differential evolution [20], and artificial bee colony (ABC) [21, 22]. Mahmoodabadi et al. [22] combined the multicrossover and the bee colony mechanism to improve the exploration capability of PSO. In [9], an elitist learning strategy, similar to the mutation operation, is developed to help the* gbest* particle jump out of the local optima.

The topological structure of the swarm has a significant effect on the performance of PSO [23–26]. Kennedy [23] pointed out that small neighborhood is fit for complex problems, while large neighborhood is used for simple problems. Parsopoulos and Vrahatis [24] integrated the benefits of the global PSO and the local PSO and then proposed a unified PSO (UPSO). Mendes et al. [25] proposed a fully informed PSO (FIPSO) in which the updating velocity depends on neighborhoods of each particle instead of* gbest* and* pbest*. Bratton and Kennedy [26] proposed a standard version of PSO (SPSO) in which a local ring topology is employed. Experimental results indicated that the local model is more effective than the global model on many test problems.

The design of learning strategies improves the performance of PSO in complex multimodal problems [27–31]. In the basic PSO, each particle learns from* gbest*. Hence, the swarm diversity loses easily in the initial evolutionary process. Zhou et al. [27] developed a random position PSO (RPPSO). In [27], if the randomly generated number is smaller than the acceptance probability, a random position is used to guide the particle. Liang et al. [28] proposed a comprehensive learning PSO (CLPSO) in which each particle can select its* pbest* or other's* pbest* as the learning exemplar according to the given probability. Li et al. [29] developed a self-learning PSO which contains four learning strategies: exploitation, jumping out, exploration, and convergence. Huang et al. [30] proposed an example-based learning PSO (ELPSO) algorithm that uses multiple global best positions as elite examples to retain the swarm diversity. Chen et al. [31] proposed a PSO with an aging leader and challengers (ALC-PSO). In [31], an aging mechanism is designed to promote a suitable leader to lead the evolution of the swarm.

Multiswarm PSO (MS-PSO) [32–36] is developed to maintain the balance of the exploration/exploitation search. In a homogenous MS-PSO, each swarm adopts a similar learning strategy. On the contrary, each swarm uses different learning strategy to implement the different search task in a heterogeneous MS-PSO. Niu et al. [32] presented a multiswarm cooperative optimizer (MCPSO) where the population consists of a master swarm and several slave swarms. Each slave swarm searches the better solution independently, while the master swarm collects the best particles from the slaves to refine the global optimization. Sun and Li [33] presented a two-swarm cooperative PSO (TCPSO) where one swarm is used to concentrate around the local optimum and accelerate the convergence, and the particles of the other one are dispersed around the search interval to keep the diversity.

Local topological structures and dynamical exemplar strategies can keep the swarm diversity to efficiently prevent the premature convergence, but the convergence rate is slow. The heterogeneous multiswarm method is powerful in balancing the local search and the global search by taking advantage of different learning strategies of the subswarms. In this method, it is important to design learning strategies which directly influence the performance of the algorithm. In order to develop efficient learning strategies, this paper analyzes the motion behavior of the swarm based on the probability characteristic of the learning parameters. Meanwhile, we point out that the probability characteristic of the learning parameters has an influence on the search space of the particles. Then, we propose a PSO with double learning patterns (PSO-DLP) to improve both the convergence rate and the accuracy of PSO. PSO-DLP adopts the master swarm and the slave swarm to obtain a good balance between the exploitation and exploration search. The master swarm is used in the exploration search, and the slave swarm is employed to carry out the exploitation search and to accelerate the convergence. The two swarms fulfill their tasks by adjusting the probability characteristics of the learning parameters. An interaction mechanism between two swarms is developed, which can help the slave swarm in jumping out of the premature convergence and improve the convergence precision of the master swarm. The simulation results show that PSO-DLP has powerful ability of global search and fast convergence speed. Experimental studies on 20 well-known benchmark functions show that the proposed PSO-DLP obtains a promising performance in terms of the accuracy and the convergence speed.

The rest of this paper is organized as follows.  Section 2 describes the basic PSO.  Section 3 presents the behavior analysis of the basic PSO.  Section 4 presents the methodologies of PSO-DLP in detail.  Section 5 provides the experimental settings and the results. Finally, Section 6 concludes this work.

## 2. Basic PSO

PSO is a population-based algorithm, which consists of a group of particles. Each particle *i* is represented by two vectors, namely, a position vector **X**_**i**_ = (*X*_*i*1_, *X*_*i*2_,…, *X*_*iD*_) and a velocity vector **V**_**i**_ = (*V*_*i*1_, *V*_*i*2_,…, *V*_*iD*_). The position of each particle in the search space is treated as a potential solution. Each particle *i* updates its velocity and position with the following equations:

(1) Vidt+1=wVidt+c1r1tPbidt−Xidt+c2r2tGbdt−Xidt

(2) Xidt+1=Xidt+Vidt+1,

where *X*_*id*_(*t* + 1) and *V*_*id*_(*t* + 1) represent the *d*th dimension of the position and the velocity of particle *i* at the (*t* + 1)th iteration. **P** **b**_**i**_ = (*Pb*_*i*1_, *Pb*_*i*2_,…, *Pb*_*iD*_) (*pbest*) is the personal best experience of the *i*th particle and **G** **b** = (*Gb*_1_, *Gb*_2_,…, *Gb*_*D*_) (*gbest*) is the group best experience found by the whole swarm. *w* is an inertia weight; *c*_1_ and *c*_2_ are acceleration coefficients reflecting the weighting of the stochastic acceleration terms that pull each particle toward* pbest* and* gbest*, respectively. Random factors *r*_1_ and *r*_2_ are two independent random numbers in the range of [0, 1].

The first item of (1) (i.e., *wV*_*id*_) is the previous velocity, which provides the necessary momentum for particles to roam around the search space. The second item (i.e., *c*_1_ *r*_1_(*Pb*_*id*_ − *X*_*id*_)), known as the “cognitive” component, represents the personal thinking of each particle. The cognitive component encourages the particles to move towards* pbest*. The third item (i.e., *c*_2_ *r*_2_(*Gb*_*d*_ − *X*_*id*_)), regarded as the “social” component, expresses the collaborative effect of the particles in finding the global optimal solution. The social component always pulls the particles to* gbest*.

## 3. Behavior Analysis of PSO

In the basic PSO, each particle toward the optimum solution is guided by the cognitive component and the social component. Therefore, proper control of these two components is very important to find the optimum solution accurately and efficiently [3]. Hence, researchers have proposed various strategies, such as the linearly varying acceleration coefficients [3], the nonlinearly varying acceleration coefficients [9–11], and the time-varying acceleration coefficients with the evolutionary state [16, 17]. In addition, Krohling [14] presented a Gaussian PSO in which random factors and acceleration coefficients are instead of the two positive random numbers generated according to the Gaussian distribution. Similar to the Gaussian PSO, Richer and Blackwell [15] introduced the Lèvy distribution to replace the uniform distribution of random factors. The previous works show that the setting of acceleration coefficients and the probability distribution of random factors can affect the performance of PSO.

In order to facilitate the analysis, the velocity updating equation needs to be transformed. Substituting (1) into (2), we obtain

(3) Xidt+1=Xidt+c1r1tPbidt−Xidt+c2r2tGbdt−Xidt+wVidt.

According to (3), we can also write

(4) Xidt+1=Xidt+wVidt+c1+c2·c1r1c1+c2+c2r2c1+c2·c1r1Pbidtc1r1+c2r2+c2r2Gbdtc1r1+c2r2−Xidt.

Let *k* = *c*_1_/(*c*_1_ + *c*_2_). Then (4) can be simplified as follows:

(5) Xidt+1=Xidt+wVidt+c1+c2kr1+1−kr2kr1Pbidtkr1+1−kr2+1−kr2Gbdtkr1+1−kr2−Xidt.

Let *D*_*f*_ = *kr*_1_ + (1 − *k*)*r*_2_ and *L*_*f*_ = *kr*_1_/(*kr*_1_ + (1 − *k*)*r*_2_); then from (5), we get

(6) Xidt+1=Xidt+c1+c2·DfLfPbidt+1−LfGbdt−Xidt+wVidt,

where *D*_*f*_ and *L*_*f*_ are functions with respect to the random factors *r*_1_, *r*_2_ and the undetermined parameter *k*. Hence *D*_*f*_ and *L*_*f*_ are correlative random variables in [0, 1]. From (6), the movement of the particles from the *t*th iteration to the (*t* + 1)th iteration can be divided into two parts. The particles firstly enter into a search space, defined by the second term of (6), and then make an inertia motion decided by the third term. Let *Pu* = *L*_*f*_ *Pb*_*id*_(*t*) + (1 − *L*_*f*_)*Gb*_*d*_(*t*), where *Pu* is a point on the line connected by *Pb*_*id*_(*t*) and *Gb*_*d*_(*t*). Given a fixed *L*_*f*_, the term (*Pu* − *X*_*id*_(*t*)) represents a line segment from *X*_*id*_(*t*) to *Pu*, namely, one-step search space. The acceleration coefficient and the variable *D*_*f*_ can influence the size of the one-step search space, and *D*_*f*_ can also decide the distribution of the location of particles in this space. The variable *L*_*f*_ is the weighting coefficient, which reflects the differences of exploitation of* pbest* and* gbest*. When *L*_*f*_ = 1, the particle learns from* pbest*; when *L*_*f*_ = 0, the particle learns from* gbest*. Variables *L*_*f*_ and *D*_*f*_ are the learning factor and the distribution factor.

In order to analyze the effect of the two factors on the one-step search space, we need to calculate the probabilistic characteristics of the factors. Considering a general situation, that is, *c*_1_ = *c*_2_ (i.e., *k* = 0.5), we obtain

(7) Lf=r1r1+r2Df=0.5r1+0.5r2.

*r*_1_ and *r*_2_ are two independent uniform random numbers in the range of [0, 1]. Hence we calculate the density function (*f*_*Lf*_(*l*_*f*_)) of the learning factor and the joint density (*f*(*d*_*f*_, *l*_*f*_)) of the learning factor and the distribution factor (see the Appendix). The density function *f*_*Lf*_(*l*_*f*_) and the joint density *f*(*d*_*f*_, *l*_*f*_) are given by

(8) fLflf=121−lf2,0≤lf<0.512lf2,0.5≤lf≤1<0.50,other

(9) fdf,lf=4df,0≤df≤1,  max⁡0,1−12df≤lf≤min⁡1,12df0,other.

Using (8) and (9), we can calculate *f*_*D*_*f*_∣*L*_*f*__(*d*_*f*_∣*l*_*f*_) the conditional probability density of *D*_*f*_ given *L*_*f*_:

(10) fDf ∣ Lfdf ∣ lf=8df1−lf2,0≤lf<12,  0≤df<121−lf8dflf2,12≤lf<1,  0≤df<12lf.

**Figure 1:**
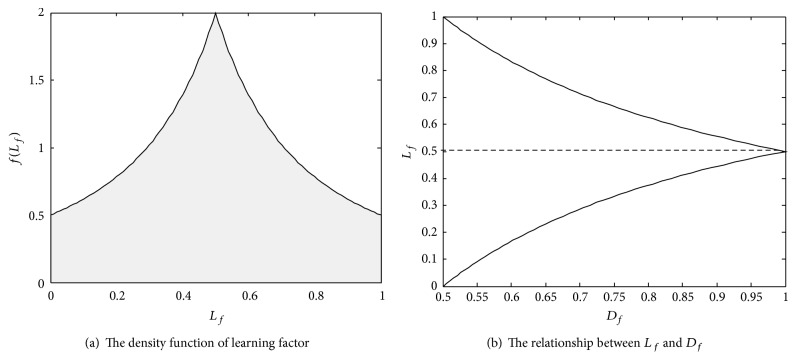
The probability characteristics of the learning parameters.

We can see from (8) that *f*(*l*_*f*_) is a unimodal function and it is symmetrical at *L*_*f*_ equal to 0.5 (Figure 1(a)). If the interval of the learning factor is divided into three smaller ones, that is, (0, 0.25), (0.25, 0.75), and (0.75, 1), the probability of the learning factor lying in the interval [0, 0.25] is the same as that in interval [0.75, 1]. When the learning factor is located in the interval [0, 0.25], we consider the particle emphasis on learning from* gbest*. If, on the contrary, the learning factor is located in the interval [0.75, 1], the particle pays attention to learning from* pbest*.

From (10), we can see that the range of the distribution factordepends on the value of the learning factor. The relationship between the learning factor and the distribution factor is shown in Figure 1(b). When the learning factor equals 0.5, the value of the distribution factor ranges from 0 to 1. When the learning factor equals 0 or 1, the value of the distribution factor ranges from 0 to 0.5. If given a fixed *Z*, *f*(*d*_*f*_∣*l*_*f*_) increases with the rising of the distribution factor. This means that the particles tend to the large value of the distribution factor.

In the basic PSO, the probability characteristics of the learning factor and the distribution factor may bring forth the clustering of the swarm. (1) During an iteration, each particle emphasizes learning from* gbest* with the probability that *P*(0 < *l*_*f*_ < 0.25) = 1/6; at the same time, the value range of the distribution factor is restricted because the learning factor is in the interval of [0, 0.25], which means that the one-step space of the particle is lessened. This situation will eventually lead to the swarm clustering move toward* gbest*. (2) The conditional probability density *f*(*d*_*f*_∣*l*_*f*_) of the distribution factor is an increasing function with the increased distribution factor, which means that the particle trends to a “long-distance flying” in the one-step space. This flying may accelerate the clustering of the particles. (3) When the learning factor equals 0.5, the value of the distribution factor falls into its maximum range from 0 to 1. But the probability of the learning factor around 0.5 is 0.182, *P*(0.5 − *δ* < *d*_*f*_ < 0.5 + *δ*) = 0.182  (*δ* = 0.05). In other words, the one-step space of the particle is shrunk with the probability of 0.818. All of the above reasons may bring about the quick clustering of the swarm.

## 4. PSO with Double Learning Patterns (PSO-DLP)

In this section, we describe PSO-DLP in detail. According to the analysis of the basic PSO, we know that the probability characteristics of the learning parameters can influence the search behavior of particles. PSO-DLP takes advantage of the probability characteristics of the leaning parameters to achieve the global search.

### 4.1. Learning Patterns

In PSO-DLP, we develop two learning patterns, called a uniform exploration and an enhanced exploitation. And two swarms, namely, a master swarm and a slave swarm, are also employed. The master swarm adopts the uniform exploration to avoid the premature convergence and the slave swarm uses the enhanced exploitation to accelerate the convergence speed.

In the uniform exploration learning, we present three novel strategies. Firstly, the learning factor and the distribution factor are independent. The value of the distribution factor varies from 0 to 1, which enlarges the search space of particles efficiently. In the basic PSO, the value of the distribution factor reaches its maximum value of 1 only when the value of the learning factor equals 0.5. Secondly, the distribution factor is subject to the uniform distribution in [0, 1], which is beneficial for preserving the swarm diversity. Thirdly, the learning factor decreases with iteration, which helps the particles in emphasizing the exploration in the earlier stage of the evolution and enhancing the convergence in the later stage. For particle *i* in the master swarm, its velocity *V*_*i*_^*m*^ is updated as follows:

(11) Vidmt+1=wVidmt+c1m+c2m·DfmLfm·Pbidmt+1−LfmGbdmt−Xidmt

(12) fLfmlfm=1,0≤lfm<10,other

(13) Lfm=Lf,maxm−Lf,maxm−Lf,minmiteritermax,

where *L*_*f*,max_^*m*^ = 1, *L*_*f*,min_^*m*^ = 0; iter  is the number of fitness evaluations (FEs); and iter_max_ is the maximum FEs defined by the user. *Gb* ^*m*^ is the* gbest *of the master swarm.

The purpose of the enhanced exploitation learning is to make the search focus on a region for refining a promising solution. In this pattern, the learning factor and the distribution factor are independent. The learning factor is a uniform random number in [0, 0.5], which can make the search concentrate around* gbest*. The distribution factor is also a random number generated in the interval of [0, 0.5] uniformly, which shrinks the search space to accelerate the convergence.

For particle *i* in the master swarm, its velocity *V*_*i*_ updates as follows:

(14) Vidst+1=wVidst+c1s+c2s·DfsLfs·Pbidst+1−LfsGbdst−Xidst

(15) fLfslfs=2,0≤lfs<0.50,other

(16) fDfsdfs=2,0≤dfs<0.50,other,

where *Gb* ^*s*^ is the* gbest *of the slave swarm.

### 4.2. Interaction between Swarms

In PSO-DLP, two learning patterns play different roles in the evolutionary process. The master swarm is used for the global search, while the slave swarm is employed for the local search. But particles in the slave swarm get easily trapped in the local optima. To improve the convergence precision of the slave swarm, the interaction between two swarms is necessary. This interaction is unidirectional and the information flow is from the master swarm to the slave swarm. Particles in the master swarm do not receive information from the slave swarms in order to keep the ability to perform the global search. When the best particle (*Gb* ^*s*^) in the slave swarm is not improved from *L* successive FEs or the fitness value of *Gb* ^*s*^ is less than the one of the best particle (*Gb* ^*m*^) in the slave swarm (the higher the fitness value, the better the performance of the particle), particles in the slave swarm can learn from the best particle in the master swarm (*Gb* ^*m*^)  (*Gb* ^*s*^ = *Gb* ^*m*^). Too small values and too large ones of *L* are not desirable, as the former tends to weaken the exploitation capability of particles in the slave swarm and the latter leads to wasting computation resources (as the slave swarm may suffer from the premature convergence). In this study, we set *L* = 50.

### 4.3. PSO-DLP Procedure

PSO-DLP is developed based on the two learning patterns and the interaction between the swarms discussed above. The pseudocode of the PSO-DLP is given in Algorithm 1. As no additional operation is introduced into PSO-DLP, the computational complexity and the storage memory are the same as the basic PSO. Therefore, PSO-DLP keeps the simplicity of the basic PSO.

## 5. Experimental Setup and Simulation Results

### 5.1. Benchmark Functions

The 20 scalable benchmark functions are used to investigate the performance of the proposed algorithm, including unimodal functions, multimodal functions, rotated functions, and shifted functions. These functions are widely adopted in [3, 8–13, 19–40]. These problems are minimization problems and the expressions of the functions are given in Table 1. The shifting and rotating methods used in the test functions are from [28, 39]. In Table 1, *M* denotes the orthogonal matrix, and *o* denotes the shifted global optimum and *f*_bias_ denotes the shifted fitness value. All functions are evaluated with 30 variables.

### 5.2. Parameter Settings for the Involved PSO Variants

For the comprehensive comparison with PSO-DLP, eight PSO variants are employed in this paper. They are PSO-W [8], HPSO [3], FIPS [25], CLPSO [28], SPSO [26], HEPSO [22], COMPSO [32], and TS-CPSO [33]. The parameter settings of each peer algorithm are extracted from the corresponding literatures and they are given in Table 2.

The swarm size is set to 40 [28] for eight algorithms expect for COMPSO. For COMPSO, the population size is set at 80, as the suggestion proposed by its author. COMPSO has four subswarms, and TS-CPSO and PSO-DLP have two subswarms. In the three algorithms, each subswarm has the same size which is set to 20. To ensure a fair comparison, the maximum number of fitness evaluations (FEs) is set at 1.00*E* + 5. Each algorithm was run 30 times independently to reduce random discrepancy.

### 5.3. Performance Metrics

In this study, we adopted the fitness mean (Fm), success rate (SR), and success performance (SP) to assess the accuracy, the reliability, and the efficiency of PSO, respectively [39]. Fm is the mean difference *f*(*x*) − *f*(*x* ^*∗*^) between the best fitness value of the algorithm and the global optimum (*x* ^*∗*^ and *x* represent the global solution and the best solution found by an algorithm, resp.). SR denotes the consistency of an algorithm to achieve the solution with a predefined *ε*. SP denotes the number of FEs required by an algorithm to solve the problem with a predefined *ε*. The Wilcoxon test [41, 42] is applied to perform pairwise comparison between PSO-DLP and its peers. The confidence level is fixed at 0.95. If the performance of PSO-DLP is better than its peer, the *h* value is denoted by “+”; if the *h* value is “ = ” or “−”, this indicates that the performance of PSO-DLP is almost the same as or is significantly worse than its peers, respectively. The average ranking can be calculated to undertake multiple comparisons [41, 42].

### 5.4. Parameter Sensitivity Analysis

The effect of parameter *L* on the performance of PSO-DLP was investigated. The seven benchmarks are selected, namely, *F*_1_, *F*_4_, *F*_7_, *F*_8_, *F*_10_, *F*_12_, *F*_14_, and *F*_17_. The *L* value of PSO-DLP was set to an integer value varying from 10 to 100.  Table 3 presents the mean values and the standard deviation obtained by PSO-DLP with different *L*.

From Table 3, we can see that the experimental results of functions *F*_1_ and *F*_4_ decrease with the increasing of *L*  because *F*_1_ and *F*_2_ are unimodal and the higher value of *L* is beneficial to the exploitation search. For multimodal functions *F*_7_, *F*_8_, *F*_10_, *F*_12_, *F*_14_, and *F*_17_, the performance of PSO-DLP tends to deteriorate when *L* is set too low (i.e., *L* = 10, 20, and 30) or too high (i.e., *L* = 90, 100). When *L* is set too low, the interaction between subswarms is overemphasized such that the slave swarm receives the best solution from the master swarm so frequently that the search of the slave swarm is not sufficient to refine the search. When *L* is set too high, the interaction between swarms is not enough such that the slave swarm may get into the premature convergence for solving multimodal functions. If the interaction interval *L* is long, the master swarm could not help the slave swarm in getting out of the local optimum. From the above experimental findings, the parameter value of *L* at 50 had the promising performance of PSO-DLP. Hence, this parameter setting was adopted in the following experiments.

### 5.5. Experimental Results and Discussions

#### 5.5.1. Comparison among the Fm Results

Table 4 presents the results of the fitness means Fm and standard deviation SD of the nine algorithms on the conventional problems, rotated problems, and shifted problems. The best results among the nine algorithms are shown in bold.

From Table 4, we obverse that PSO-DLP obtains the best searching accuracy in the eight conventional problems, except for the function *F*_5_. To be specific, PSO-DLP finds the global optima of *F*_1_, *F*_6_, *F*_7_, and *F*_8_. In addition, PSO-DLP is the only algorithm to achieve the accuracy level of 10^−19^ for the function *F*_4_. For the function *F*_5_, COMPSO obtains the best result and PSO-DLP ranks the third.

The functions in group 2 (*F*_10_–*F*_14_) are multimodal functions with coordinate rotations. As can be seen from Table 3, all the algorithms perform worse for the rotated functions than the unrotated counterparts. PSO-DLP performs the best on functions *F*_10_, *F*_11_, and *F*_14_, that is, 3 out of 5 functions, in terms of the Fm. For function *F*_13_, TS-CPSO obtains the best fitness value, followed by PSO-DLP.

From Table 4, we observe that all involved algorithms cannot find the global optima for shifted problems (*F*_15_–*F*_18_) and complex problems (*F*_19_-*F*_20_), except for function 15. Function 15 is an easy shifted problem; only PSO-DLP achieves the global optima (Fm = 0). PSO-DLP surpasses all other algorithms on the four functions. This implies that PSO-DLP is the least sensitive one to the shifted problem. Complex problems are rotating and shifting operations (*F*_19_-*F*_20_). PSO-DLP obtains the best solutions on complex functions.

#### 5.5.2. Comparison of Nonparametric Statistical Test Results

The Wilcoxon test results between PSO-DLP and peers are reported in Table 4. From the test results, PSO-DLP significantly outperforms all other algorithms on functions *F*_2_, *F*_3_, *F*_4_, *F*_8_, *F*_9_, *F*_10_, *F*_14_, *F*_15_, *F*_16_, *F*_17_, *F*_18_, and *F*_20_. COMPSO performs the best on function *F*_5_. For function *F*_5_, PSO-DLP and COMPSO do not show significant difference according to their statistical test values. PSO-DLP obtains the global solution on functions *F*_6_ and *F*_7_. For functions *F*_6_ and *F*_7_, the performance of HEPSO, TS-CPSO, and CLPSO is almost the same as that of PSO-DLP as the Wilcoxon test results show.

To compare the performance of multiple algorithms, we used the average ranking of the Friedman test to evaluate the effectiveness of PSO-DLP comprehensively. The algorithm with the better convergence accuracy is assigned to a smaller rank value.  Table 5 shows the average ranking of nine PSO variants on functions *F*_1_–*F*_20_. According to the result of the average ranking, nine algorithms can be sorted into the following order: PSO-DLP, HEPSO, COMPSO, CLPSO, HPSO, TS-CPSO, PSO-W, SPSO, and FIPS. PSO-DLP obtains the best performance.

#### 5.5.3. Comparisons of the SR Value and the SP Value

The SR values and the SP values of all algorithms are shown in Table 6. If an algorithm is unable to completely solve the problems, then the value of SR is set at zero (i.e., SR = 0) and the value of SP is assigned an infinity value (i.e., SP =“inf”). As shown in Table 6, we observe that PSO-DLP has more superior search reliability among the compared peers. It can completely solve all conventional problems and rotated problems except for functions *F*_9_, *F*_13_, and *F*_14_. For functions *F*_9_, *F*_13_, and *F*_14_, none of the compared algorithms can solve the problem completely at the predefined *ε* and PSO-DLP can achieve the best result on functions *F*_9_ and *F*_14_ from Table 3. For the shifted problems, PSO-DLP completely solves four out of six functions at the predefined *ε*. Meanwhile, none of the algorithms are able to solve functions *F*_19_ and *F*_20_. Nevertheless, PSO-DLP can obtain the smallest Fm value for functions *F*_19_ and *F*_20_.

**Figure 2:**
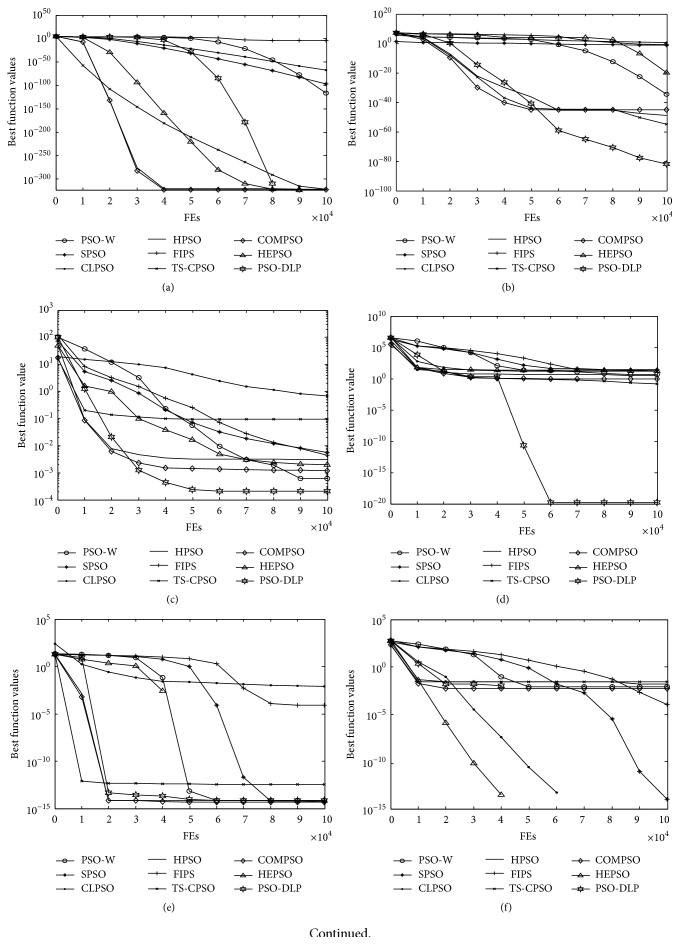
Convergence curves of test functions. (a) *F*_1_, (b) *F*_2_, (c) *F*_3_, (d) *F*_4_, (e) *F*_5_, (f) *F*_6_, (g) *F*_7_, (h) *F*_8_, (i) *F*_9_, and (j) *F*_10_. Convergence curves of test functions. (k) *F*_11_, (l) *F*_12_, (m) *F*_13_, (n) *F*_14_, (o) *F*_15_, (p) *F*_16_, (q) *F*_17_, (r) *F*_18_, (s) *F*_19_, and (t) *F*_20_.

As reported in Table 6, PSO-DLP can get seven best SP values in conventional problems and rotated problems. The results show that PSO-DLP costs the least computation to solve these problems at the predefined *ε*.  Figure 2 shows the convergence curves of the nine PSO algorithms on all functions. It can be seen that the convergence curves for functions *F*_2_, *F*_4_, *F*_10_, *F*_11_, and *F*_20_ (Figures 2(b), 2(d), 2(j), 2(k), and 2(t)) show that the PSO-DLP has the competitive convergence speed among its peers. For functions *F*_9_, *F*_17_, and *F*_18_ (Figures 2(i), 2(q), and 2(r)), PSO-DLP converges slowly at the beginning and achieves with fast convergence in later stage of the evolution. Particularly, the convergence speed of PSO-DLP in functions *F*_6_, *F*_7_, and *F*_8_ (Figures 2(f), 2(g), and 2(h)) is significantly faster than its peers in the middle stage of the evolution. Overall, PSO-DLP has better control of the convergence speed and accuracy than the involved PSO variants.

#### 5.5.4. Effect of Different Strategies

The proposed PSO-DLP employs two learning patterns: the uniform exploration leaning (UEL) and the enhanced exploitation learning (EEL). To investigate the effects of them, we studied the performance of PSO-W, PSO with UEL (PSO-UEL), PSO with EEL (PSO-EEL), and the complete PSO-DLP. PSO-UEL and PSO-EEL adopt one swarm so that there is no interaction between the subswarms. The Fm values of all involved algorithms are presented in Table 7.

From Table 7, PSO-UEL achieves better results than PSO-W on 15 functions, and it successfully solves multimodal functions *F*_7_ and *F*_8_, which means that PSO-UEL has strong exploration ability. Meanwhile, we observe that PSO-EEL performed well in unimodal functions (*F*_1_, *F*_2_, *F*_3_, and *F*_15_) and Rosenbrock functions (*F*_4_, *F*_10_, and *F*_16_), but it fails to solve the other multimodal functions. This observation means that PSO-EEL is easier to be trapped in the local optima for multimodal functions. By integrating the two learning patterns, PSO-DLP obtains excellent performance.

#### 5.5.5. Performance Comparisons between PSO-DLP and Other Evolutionary Algorithms

In this section, we compare PSO-DLP with the covariance matrix adaptation evolution strategy (CMA-ES) [43], self-adaptive DE (SaDE) [44], and adaptive DE with optional external archive (JADE) [45]. The CMA-ES algorithm is a powerful stochastic optimization technique, where the multivariate normal distribution has a mean and a covariance matrix constantly updated during the evolutionary process. Differential evolution (DE) has been shown to be an efficient stochastic search algorithm for solving optimization problems. SaDE provides a mutation strategy pool, where the strategy is adaptively selected according to its previous performance. The JADE is another adaptive DE-variant, where a novel mutation strategy is developed and an external archive is employed to store information of progress direction. Simulation results showed that JADE has competitive performances.

Twenty benchmark functions as presented in Table 1 are used to test the performance of four algorithms. The parameter values of CMA-ES and DEs are selected according to the recommendations of their respective references, as listed in Table 3. The maximum number of fitness evaluations is set at 1.00*E* + 5 and population size *N* is set at 40 (*N* = (4 + ⌊3log⁡(*D*)⌋) for CMA-ES) for all algorithms. All functions are tested on 30 dimensions (*D* = 30). All algorithms are run independently 30 times to ensure the fair assessment.

The mean (Fm) and the standard deviation (SD) of the results obtained by each algorithm for all algorithms are summarized in Table 8. From Table 8, we observed that PSO-DLP has better performance on eleven functions and has the same performance with DEs or CMA-ES on six functions. From the final rank, we can obtain the order of the involved algorithms: PSO-DLP, JADE, CMA-ES, and SADE. PSO-DLP has competitive performance among these algorithms on the tested functions.

## 6. Conclusion

In this paper we study the motion behavior of the swarm and point out that the probabilistic characteristics of the learning factor and the distribution factor in the updating equation can affect the searching behavior of particles. We develop a Particle Swarm Optimization with double learning patterns (PSO-DLP), which uses two swarms with different searching ability and the interaction mechanism between swarms to control exploration and exploitation searches. In the PSO-DLP, the master swarm encourages the exploration process while the slave swarm concentrates on a small region in order to find its best local optimum easily and accelerate its convergence. The two swarms fulfill their searching tasks by adopting two different learning patterns in which the searching behavior is controlled by adjusting the probabilistic characteristics of the learning factor and the distribution factor.

The significant feature of PSO-DLP is that it has a better balance between high precision and fast convergence. Another attractive property of PSO-DLP is that it employs the probabilistic characteristics of the learning parameters to control the swarm diversity so that it does not need any additional operation.

In our future work, we will study the effect of the topological structure of the uniform exploration learning pattern. And PSO-DLP will be extended to the image segmentation and multiobjective problems.

## Figures and Tables

**Algorithm 1:**
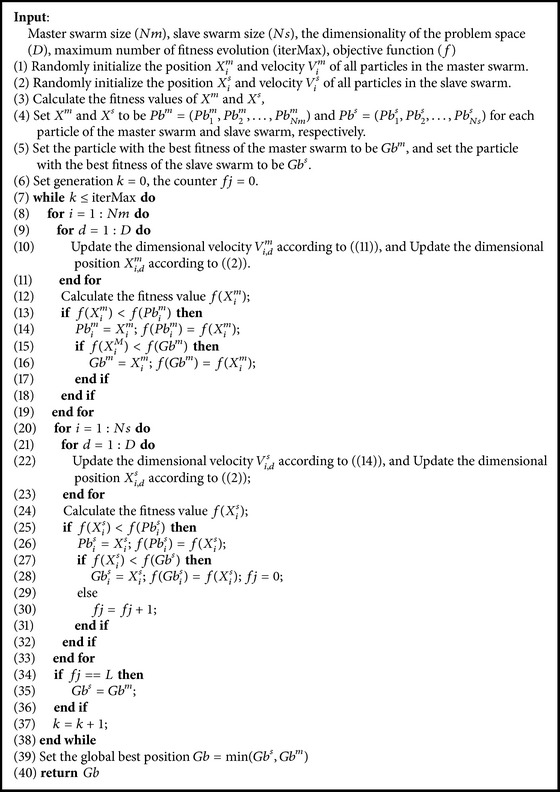
Particle swarm optimization with double learning patterns.

**Table 1:** Benchmark functions used in this paper.

Number	Description and expression	Search space	*ε*	*F*(*X* ^*∗*^)
Group 1: conventional problems				
*F*_1_	Sphere *F*_1_(*x*) = ∑_*i*=1_^*D*^ *x*_*i*_^2^	[−100, 100]^*D*^	10^−6^	0
*F*_2_	Schwefel's function 1.2 *F*_2_(*x*) = ∑_*i*=1_^*D*^(∑_*k*=1_^*i*^ *x*_*k*_)^2^	[−100, 100]^*D*^	10^−6^	0
*F*_3_	Noise quadric *F*_3_(*x*) = ∑_*i*=1_^*D*^ *ix*_*i*_^4^ + random(0,1)	[−1.28, 1.28]^*D*^	10^−2^	0
*F*_4_	Rosenbrock *F*_4_ = ∑_*i*=1_^*D*^[100(*x*_*i*+1_ − *x*_*i*_^2^)^2^ + (*x*_*i*_ − 1)^2^]	[−10, 10]^*D*^	10^−2^	0
*F*_5_	Ackley $F_{5}(x)=-20\exp\left(-0.2\sqrt{(\sum_{i=1}^{D}x_{i}^{2})/D}\right)-\exp\left((\sum_{i=1}^{D}cos(2\pi x_{i}))/D\right)+20+e$	[−32.768, 32.768]^*D*^	10^−6^	0
*F*_6_	Griewank $F_{6}(x)=\sum_{i=1}^{D}x_{i}^{2}/4000-\prod_{i=1}^{D}\cos\left(x_{i}/\sqrt{i}\right)+1$	[−600, 600]^*D*^	10^−6^	0
*F*_7_	Rastrigin *F*_7_(*x*) = ∑_*i*=1_^*D*^(*x*_*i*_^2^ − 10cos⁡(2*πx*_*i*_) + 10)	[−5.12, 5.12]^*D*^	10^−6^	0
*F*_8_	Noncontinuous Rastrigin *F*_8_(*x*) = *F*_7_(*y*) (*y*_*i*_ = {*x*_*i*_, |*x*_*i*_| < 0.5; round(2*x*_*i*_)/2, |*x*_*i*_ | ≥ 0.5})	[−5.12, 5.12]^*D*^	10^−6^	0
*F*_9_	Expanded Schaffer *F*_9_(*x*) = ∑_*i*=1_^*D*−1^ *y*(*x*_*i*_, *x*_*i*+1_) + *y*(*x*_*D*_, *x*_1_) $y(u,v)=0.5+(\sin^{2}\left(\sqrt{u^{2}+v^{2}}\right)-0.5)/(1+0.001{\left(u^{2}+v^{2})\right)}^{2}$	[−100, 100]^*D*^	10^−6^	0

Group 2: rotated problems				
*F*_10_	Rotated Rosenbrock *F*_10_(*x*_*i*_) = *F*_4_(*q*_*i*_), *q*_*i*_ = *M∗x*_*i*_, *M* is an orthogonal matrix	[−10, 10]^*D*^	10	0
*F*_11_	Rotated Ackley *F*_11_(*x*_*i*_) = *F*_5_(*q*_*i*_), *q*_*i*_ = *M∗x*_*i*_	[−32.768, 32.768]^*D*^	10	0
*F*_12_	Rotated Griewank *F*_12_(*x*_*i*_) = *F*_6_(*q*_*i*_), *q*_*i*_ = *M∗x*_*i*_	[−600, 600]^*D*^	10	0
*F*_13_	Rotated Rastrigin *F*_13_(*x*_*i*_) = *F*_7_(*q*_*i*_), *q*_*i*_ = *M∗x*_*i*_	[−5.12, 5.12]^*D*^	10	0
*F*_14_	Rotated noncontinuous Rastrigin *F*_14_(*x*_*i*_) = *F*_8_(*q*_*i*_), *q*_*i*_ = *M∗x*_*i*_	[−5.12, 5.12]^*D*^	10	0

Group 3: shifted problems				
*F*_15_	Shifted Sphere *F*_15_(*x*_*i*_) = *F*_1_(*q*_*i*_) + *f*_bias1_, *q*_*i*_ = *x*_*i*_ − *o*, *f*_bias1_ = −450	[−100, 100]^*D*^	10^−6^	−450
*F*_16_	Shifted Rosenbrock *F*_16_(*x*_*i*_) = *F*_4_(*q*_*i*_) + *f*_bias2_, *q*_*i*_ = *x*_*i*_ − *o*, *f*_bias2_ = 390	[−10, 10]^*D*^	10^−6^	390
*F*_17_	Shifted Rastrigin *F*_17_(*x*_*i*_) = *F*_7_(*q*_*i*_) + *f*_bias4_, *q*_*i*_ = *x*_*i*_ − *o*, *f*_bias4_ = −330	[−5.12, 5.12]^*D*^	10^−6^	−330
*F*_18_	Shifted non-Rastrigin *F*_18_(*x*_*i*_) = *F*_8_(*q*_*i*_) + *f*_bias5_, *q*_*i*_ = *x*_*i*_ − *o*, *f*_bias5_ = −330	[−5.12, 5.12]^*D*^	10^−6^	−330
*F*_19_	Shifted rotated Ackley's function with global optimum on bounds	[−32.76, 32.76]^*D*^	10^−6^	−140
*F*_20_	Shifted rotated Rastrigin's function	[−5.12, 5.12]^*D*^	10^−6^	−330

**Table 2:** Parameter settings of algorithms used in the comparisons.

Algorithm	Year	Population topology	Parameter settings
PSO-LDIW	1998	Fully connected	*ω*: 0.9–0.4, *c*_1_ = *c*_2_ = 2.0
HPSO	2004	Fully connected	*ω*: 0.9–0.4, *c*_1_: 2.5–0.5, *c*_2_: 0.5–2.5
FIPSO	2004	Local Ring	*λ* = 0.729, Σ*c*_*i*_ = 4.1
CLPSO	2006	Comprehensive learning	*ω*: 0.9–0.4, *c* = 2.0, *m* = 7
SPSO	2007	Local Ring	*ω* = 0.729, *c*_1_ = *c*_2_ = 1.193
COMPSO	2007	Multiswarm (fully connected)	*ω*: 0.9–0.4, *c*_1_^*s*^ = *c*_2_^*s*^ = 2.05, *c*_1_^*M*^ = *c*_2_^*M*^ = *c*_3_^*M*^ = 2
HEPSO	2014	Fully connected	*ω*: 0.9–0.4, *c*_1_: 2.5−0.5, *c*_2_: 0.5−2.5, *Pc* = 0.95, *Pb* = 0.02
TS-COMPSO	2014	Multiswarm (fully connected and local ring)	*ω* ^*M*^ = 0.9, *c*_1_^*s*^ = *c*_2_^*s*^ = 3.4, *c*_1_^*M*^ = *c*_2_^*M*^ = *c*_3_^*M*^ = 4.5
CMA-ES	2007	—	*σ* = 0.25, *μ* = (4 + ⌊2log⁡(*N*)⌋)/2
SADE	2009	—	*F* ~ *N*(CR, 0.3), CR ~ *N*(CR_*m*_, 0.1), CR_0_ = 0.5, *LP* = 50
JADE	2009	—	*p* = 0.05, *c* = 0.1
PSO-DLP	—	Multiswarm (fully connected)	*ω*: 0.9–0.3, *c*_1_^*s*^ = *c*_2_^*s*^ = *c*_1_^*m*^ = *c*_2_^*m*^ = 2.0, *L* = 50

**Table 3:** Effects of the parameter *L* on PSO-DLP in 30-D.

	Fm	*L* = 10	*L* = 20	*L* = 30	*L* = 40	*L* = 50	*L* = 60	*L* = 70	*L* = 80	*L* = 90	*L* = 100
*F*_1_	SD	2.80*E* − 191	**0.00E + 00**	**0.00E + 00**	**0.00E + 00**	**0.00E + 00**	**0.00E + 00**	**0.00E + 00**	**0.00E + 00**	**0.00E + 00**	**0.00E + 00**
	Fm	0.00*E* + 00	**0.00E + 00**	**0.00E + 00**	**0.00E + 00**	**0.00E + 00**	**0.00E + 00**	**0.00E + 00**	**0.00E + 00**	**0.00E + 00**	**0.00E + 00**

*F*_4_	SD	7.97*E* − 17	7.57*E* − 17	8.07*E* − 18	7.97*E* − 17	**2.01E − 19**	3.04*E* − 19	7.97*E* − 17	7.97*E* − 17	8.27*E* − 17	8.05*E* − 17
	Fm	1.17*E* − 17	2.10*E* − 17	3.05*E* − 18	2.00*E* − 17	**6.19E − 19**	2.33*E* − 19	2.01*E* − 17	1.02*E* − 17	2.31*E* − 17	5.01*E* − 17

*F*_7_	SD	5.90*E* − 13	4.76*E* − 14	3.55*E* − 16	1.06*E* − 15	**0.00E + 00**	**0.00E + 00**	**0.00E + 00**	**0.00E + 00**	1.59*E* − 14	1.17*E* − 14
	Fm	1.28*E* − 12	1.04*E* − 13	7.94*E* − 16	2.38*E* − 15	**0.00E + 00**	**0.00E + 00**	**0.00E + 00**	**0.00E + 00**	2.43*E* − 14	7.53*E* − 15

*F*_8_	SD	1.93*E* − 13	6.82*E* − 14	2.84*E* − 15	**0.00E + 00**	**0.00E + 00**	**0.00E + 00**	**0.00E + 00**	**0.00E + 00**	3.19*E* − 15	4.28*E* − 15
	Fm	2.67*E* − 13	1.48*E* − 13	4.08*E* − 15	**0.00E + 00**	**0.00E + 00**	**0.00E + 00**	**0.00E + 00**	**0.00E + 00**	7.14*E* − 15	1.56*E* − 15

*F*_10_	SD	1.71*E* + 00	1.71*E* + 00	1.19*E* + 00	7.46*E* − 01	**2.00E − 01**	2.01*E* − 01	2.87*E* − 01	1.72*E* + 00	1.09*E* + 00	1.94*E* + 00
	Fm	1.10*E* + 00	2.10*E* + 00	1.54*E* + 00	1.53*E* − 01	**2.83E − 01**	2.93*E* − 01	1.86*E* − 01	1.55*E* + 00	1.72*E* + 00	1.91*E* + 00

*F*_12_	SD	7.90*E* − 03	7.90*E* − 03	5.40*E* − 03	7.40*E* − 03	**3.70E − 03**	**3.70E − 03**	1.08*E* − 02	4.90*E* − 03	6.94*E* − 03	5.91*E* − 03
	Fm	5.11*E* − 03	1.39*E* − 03	8.60*E* − 03	7.00*E* − 03	**5.23E − 03**	**5.23E − 03**	1.01*E* − 02	7.00*E* − 03	9.60*E* − 03	5.70*E* − 03

*F*_14_	SD	3.48*E* + 01	3.56*E* + 01	3.02*E* + 01	2.65*E* + 01	**2.55E + 01**	**2.55E + 01**	2.92*E* + 01	2.98*E* + 01	2.56*E* + 01	2.60*E* + 01
	Fm	4.02*E* + 00	8.08*E* + 00	7.22*E* + 00	4.60*E* + 00	**1.34E + 01**	1.54*E* + 01	5.97*E* + 00	3.56*E* + 00	6.58*E* + 00	9.87*E* + 00

*F*_17_	SD	5.68*E* − 14	5.11*E* − 14	5.11*E* − 14	5.11*E* − 14	**1.27E − 14**	1.54*E* − 14	1.97*E* − 14	3.97*E* − 14	4.54*E* − 14	3.41*E* − 14
	Fm	0.00*E* + 00	1.27*E* − 14	1.27*E* − 14	1.27*E* − 14	**1.48E − 13**	1.55*E* − 14	1.57*E* − 14	1.55*E* − 14	1.55*E* − 14	1.27*E* − 14

**Table 4:** Experimental result comparisons among nine PSOs.

		PSO-W	SPSO	CLPSO	HPSO	FIPS	HEPSO	TS-CPSO	COMPSO	PSO-DLP
*F*_1_	Fm	1.34*E* − 117	8.86*E* − 97	3.35*E* − 87	5.87*E* − 322	5.66*E* − 05	**0.00E + 00**	5.92*E* − 322	1.48*E* − 323	**0.00E + 00**
	SD	2.16*E* − 116	1.86*E* − 96	5.37*E* − 87	2.18*E* − 322	9.24*E* − 06	**0.00E + 00**	4.09*E* − 322	1.87*E* − 323	**0.00E + 00**
	*h*	+	+	+	=	+	+	=	=	

*F*_2_	Fm	1.41*E* − 45	5.29*E* − 02	4.48*E* − 07	7.61*E* − 53	1.56*E* − 02	4.81*E* − 43	3.96*E* − 09	2.92*E* − 47	**3.00E − 86**
	SD	1.47*E* − 45	3.47*E* − 02	2.26*E* − 07	1.68*E* − 52	8.60*E* − 02	2.48*E* − 43	5.33*E* − 09	5.89*E* − 47	**4.25E − 86**
	*h*	+	+	+	+	+	+	+	+	

*F*_3_	Fm	2.34*E* − 03	5.62*E* − 03	3.50*E* − 03	3.10*E* − 03	4.50*E* − 03	1.61*E* − 03	9.43*E* − 03	1.21*E* − 03	**2.05E − 04**
	SD	9.83*E* − 04	1.61*E* − 03	8.73*E* − 04	2.40*E* − 03	1.30*E* − 03	6.15*E* − 04	4.80*E* − 03	4.60*E* − 04	**1.24E − 05**
	*h*	+	+	+	+	+	+	+	+	

*F*_4_	Fm	2.53*E* + 01	2.25*E* + 01	1.40*E* + 01	2.89*E* + 00	2.02*E* + 01	1.36*E* + 01	7.71*E* − 02	9.806*E* − 01	**2.01E − 19**
	SD	1.81*E* + 00	3.76*E* + 00	3.50*E* + 00	4.03*E* + 00	2.30*E* − 01	2.194*E* + 00	3.87*E* − 02	2.19*E* + 00	**6.19E − 19**
	*h*	+	+	+	+	+	+	+	+	

*F*_5_	Fm	7.11*E* − 15	5.68*E* − 15	7.10*E* − 15	7.10*E* − 15	7.54*E* − 05	**0.00E + 00**	3.34*E* − 13	4.97*E* − 15	7.11*E* − 15
	SD	0.00*E* + 00	1.94*E* − 15	0.00*E* + 00	0.00*E* + 00	2.47*E* − 06	**0.00E + 00**	3.45*E* − 13	1.94*E* − 15	0.00*E* + 00
	*h*	=	=	=	=	=	=	+	**=**	

*F*_6_	Fm	7.39*E* − 03	1.04*E* − 14	**0.00E + 00**	1.47*E* − 02	1.02*E* − 04	**0.00E + 00**	2.65*E* − 02	5.30*E* − 03	**0.00E + 00**
	SD	1.05*E* − 02	1.47*E* − 14	**0.00E + 00**	2.08*E* − 02	5.19*E* − 05	**0.00E + 00**	1.46*E* − 02	7.30*E* − 03	**0.00E + 00**
	*h*	+	+	+	+	+	**=**	+	+	

*F*_7_	Fm	2.12*E* + 01	1.31*E* + 01	1.15*E* − 14	1.50*E* − 12	8.98*E* + 02	**0.00E + 00**	1.42*E* − 15	1.65*E* + 01	**0.00E + 00**
	SD	1.02*E* + 01	1.25*E* + 01	3.63*E* − 15	3.20*E* − 12	8.17*E* + 01	**0.00E + 00**	1.48*E* − 15	3.19*E* + 00	**0.00E + 00**
	*h*	+	+	+	+	+	=	=	+	

*F*_8_	Fm	2.37*E* + 01	3.97*E* + 01	4.85*E* − 10	2.00*E* − 01	7.36*E* + 01	9.93*E* − 13	1.20*E* + 00	1.34*E* + 01	**0.00E + 00**
	SD	1.45*E* + 01	4.48*E* + 00	2.44*E* − 10	4.47*E* − 01	1.42*E* + 01	5.67*E* − 13	1.09*E* + 00	1.34*E* + 00	**0.00E + 00**
	*h*	+	+	+	+	+	=	+	+	

*F*_9_	Fm	1.27*E* + 00	2.36*E* + 00	2.59*E* + 00	1.25*E* + 00	9.07*E* + 00	1.31*E* + 00	7.03*E* + 00	2.18*E* + 00	**6.87E − 01**
	SD	4.43*E* − 01	5.21*E* − 01	5.53*E* − 01	1.75*E* − 01	**9.10E − 02**	3.33*E* − 01	1.43*E* − 01	4.36*E* − 01	3.60*E* − 01
	*h*	+	+	+	+	+	+	+	+	

*F*_10_	Fm	2.51*E* + 01	2.55*E* + 01	2.09*E* + 01	2.57*E* + 01	2.17*E* + 01	1.35*E* + 01	1.70*E* + 01	9.65*E* + 00	**2.00E − 01**
	SD	2.14*E* + 01	1.48*E* + 01	4.05*E* + 01	2.79*E* + 01	**4.94E − 02**	7.16*E* + 00	1.80*E* + 00	5.14*E* + 00	2.83*E* − 01
	*h*	+	+	+	+	+	+	+	+	

*F*_11_	Fm	1.34*E* + 00	**7.11E − 15**	1.05*E* − 13	1.20*E* + 00	7.56*E* − 05	3.61*E* − 12	1.92*E* + 00	1.48*E* + 00	**7.11E − 15**
	SD	3.14*E* − 01	**0.00E + 00**	2.13*E* − 14	7.42*E* − 01	3.85*E* − 06	8.02*E* − 12	2.97*E* − 01	5.52*E* − 01	**0.00E + 00**
	*h*	+	=	+	+	+	+	+	+	

*F*_12_	Fm	3.93*E* − 02	1.30*E* − 06	8.13*E* − 10	4.12*E* − 02	6.03*E* − 05	**0.00E + 00**	1.28*E* − 02	2.16*E* − 02	3.70*E* − 03
	SD	3.81*E* − 02	2.88*E* − 06	1.01*E* − 11	2.30*E* − 02	2.81*E* − 06	**0.00E + 00**	1.36*E* − 02	3.03*E* − 02	5.23*E* − 03
	*h*	+	−	−	+	−	** − **	+	+	

*F*_13_	Fm	5.17*E* + 01	5.59*E* + 01	3.46*E* + 01	5.51*E* + 01	1.59*E* + 02	2.89*E* + 01	**2.63E + 01**	4.29*E* + 01	2.87*E* + 01
	SD	2.25*E* + 01	1.80*E* + 00	3.69*E* + 00	10.0*E* + 00	9.76*E* + 00	5.33*E* + 00	**3.02E + 00**	7.42*E* + 00	3.68*E* + 00
	*h*	+	+	+	+	+	+	−	+	

*F*_14_	Fm	4.05*E* + 01	5.46*E* + 01	3.77*E* + 01	3.78*E* + 01	1.15*E* + 02	2.78*E* + 01	3.22*E* + 01	4.48*E* + 01	**2.55E + 01**
	SD	1.48*E* + 01	8.26*E* + 00	5.44*E* + 00	5.40*E* + 00	13.0*E* + 01	3.74*E* + 00	8.07*E* + 00	8.70*E* + 00	**1.34E + 01**
	*h*	+	+	+	+	+	+	+	+	

*F*_15_	Fm	5.68*E* − 14	4.48*E* − 14	5.68*E* − 10	4.54*E* − 14	2.40*E* + 01	5.01*E* − 07	5.68*E* − 14	3.41*E* − 14	**0.00E + 00**
	SD	0.00*E* + 00	3.21*E* − 14	1.23*E* − 10	2.54*E* − 14	2.72*E* + 01	3.23*E* − 07	1.01*E* − 14	3.11*E* − 14	**0.00E + 00**
	*h*	+	+	+	+	+	+	+	+	

*F*_16_	Fm	1.04*E* + 01	3.03*E* + 01	1.25*E* + 01	2.10*E* + 00	2.02*E* + 01	4.87*E* + 01	1.40*E* − 01	4.26*E* + 00	**1.44E − 09**
	SD	2.11*E* + 00	7.93*E* + 00	8.10*E* + 00	4.69*E* + 00	2.64*E* − 01	1.63*E* + 00	2.37*E* − 01	5.20*E* + 00	**3.21E − 09**
	*h*	+	+	+	+	+	+	+	+	

*F*_17_	Fm	2.41*E* + 01	1.86*E* + 01	5.68*E* − 14	3.58*E* + 00	1.25*E* + 02	8.36*E* + 00	4.58*E* + 01	1.51*E* + 01	**1.27E − 14**
	SD	1.19*E* + 01	1.02*E* + 01	1.63*E* − 14	4.19*E* + 00	3.26*E* + 01	6.20*E* + 00	2.25*E* + 01	3.92*E* + 00	**1.48E − 15**
	*h*	+	+	+	+	+	+	+	+	

*F*_18_	Fm	2.51*E* + 01	1.84*E* + 01	8.68*E* − 8	4.60*E* + 00	1.09*E* + 02	5.75*E* + 00	3.56*E* + 02	1.54*E* + 01	**4.55E − 14**
	SD	1.32*E* + 01	8.73*E* + 00	1.83*E* − 8	3.04*E* + 00	2.63*E* + 01	2.29*E* + 00	4.65*E* + 02	5.77*E* + 00	**2.54E − 14**
	*h*	+	+	+	+	+	+	+	+	

*F*_19_	Fm	2.06*E* + 01	2.08*E* + 01	2.07*E* + 01	2.06*E* + 01	2.09*E* + 01	2.05*E* + 01	**2.04E + 01**	2.08*E* + 01	**2.04E + 01**
	SD	6.74*E* − 01	7.57*E* − 01	4.96*E* − 01	6.74*E* − 01	2.20*E* − 01	4.03*E* − 01	2.61*E* − 01	6.51*E* − 02	**6.25E − 02**
	*h*	+	+	+	+	+	+	=	+	

*F*_20_	Fm	7.78*E* + 01	9.67*E* + 01	6.03*E* + 01	1.89*E* + 02	4.20*E* + 01	8.20*E* + 01	4.01*E* + 01	6.72*E* + 01	**3.68E + 01**
	SD	1.38*E* + 01	2.09*E* + 01	9.64*E* + 00	9.88*E* + 00	8.08*E* + 00	2.25*E* + 01	2.13*E* + 01	1.34*E* + 01	**2.81E + 00**
	*h*	+	+	+	+	+	+	+	+	

**Table 5:** Average rankings achieved by Friedman test for the nine PSO algorithms.

Algorithm	Ranking
PSO-W	5.85 (8)
SPSO	6.4 (7)
CLPSO	4.65 (4)
HPSO	5.5 (5)
FIPS	7.3 (9)
HEPSO	3.4 (2)
TS-CPSO	5.15 (6)
COMPSO	4.45 (3)
PSO-DLP	**1.5 (**1**)**

**Table 6:** Comparisons of the SR and the SP among nine PSOs.

		PSO-W	SPSO	CLPSO	HPSO	FIPS	HEPSO	TS-CPSO	COMPSO	PSO-DLP
*F*_1_	SR	100	100	100	**100**	56.00	**100**	**100**	**100**	**0.00E + 00**
	SP	2.17*E* + 03	5.23*E* + 04	6.44*E* + 04	3.45*E* + 03	INF	3.12*E* + 04	2.23*E* + 03	2.47*E* + 03	**2.04E + 03**

*F*_2_	SR	100	0.00	93.33	100	0.00	100	100	100	100
	SP	8.23*E* + 04	INF	8.89*E* + 04	3.68*E* + 04	INF	3.12*E* + 04	7.15*E* + 04	**1.12E + 04**	2.54*E* + 04

*F*_3_	SR	100	86.66	90.00	100	83.3	100	90.00	100	**100**
	SP	6.23*E* + 04	7.48*E* + 04	4.16*E* + 04	3.17*E* + 04	8.33*E* + 04	3.92*E* + 04	3.54*E* + 04	2.57*E* + 04	**1.83E + 04**

*F*_4_	SR	0	0	0	0	0	0	0	0	**100**
	SP	INF	INF	INF	INF	INF	INF	INF	INF	**4.62E + 04**

*F*_5_	SR	**100**	**100**	**100**	**100**	**100**	**100**	**100**	**100**	**100**
	SP	4.21*E* + 04	6.34*E* + 04	5.84*E* + 04	2.12*E* + 04	6.16*E* + 04	5.52*E* + 04	9.23*E* + 03	9.85*E* + 03	**1.51E + 04**

*F*_6_	SR	0	**100**	**100**	0	0	**100**	0	0	**100**
	SP	INF	7.56*E* + 04	3.81*E* + 04	INF	INF	**2.35E + 04**	INF	INF	4.15*E* + 04

*F*_7_	SR	0	0	**100**	**100**	0	**100**	**100**	0	**100**
	SP	INF	INF	6.14*E* + 04	5.52*E* + 04	INF	4.36*E* + 04	5.74*E* + 04	INF	**4.26E + 04**

*F*_8_	SR	0	0	**100**	0	0	**100**	0	0	**100**
	SP	INF	INF	9.78*E* + 04	INF	INF	8.94*E* + 04	INF	INF	**4.85E + 04**

*F*_9_	SR	0	0	0	0	0	0	0	0	0
	SP	INF	INF	INF	INF	INF	INF	INF	INF	INF

*F*_10_	SR	0	0	0	0	0	0	0	80.00	**100**
	SP	INF	INF	INF	INF	INF	INF	INF	8.15*E* + 04	**3.27E + 04**

*F*_11_	SR	**100**	**100**	**100**	**100**	**100**	**100**	**100**	**100**	**100**
	SP	3.85*E* + 04	4.27*E* + 04	3.27*E* + 04	**1.32E + 04**	6.28*E* + 04	4.87*E* + 04	3.75*E* + 04	3.27*E* + 04	5.63*E* + 04

*F*_12_	SR	**100**	**100**	**100**	**100**	**100**	**100**	**100**	**100**	**100**
	SP	3.61*E* + 04	5.78*E* + 04	2.57*E* + 04	3.15*E* + 04	5.27*E* + 04	3.16*E* + 04	2.10*E* + 04	2.18*E* + 04	1.36*E* + 04

*F*_13_	SR	0	0	0	0	0	0	0	0	0
	SP	INF	INF	INF	INF	INF	INF	INF	INF	INF

*F*_14_	SR	0	0	0	0	0	0	0	0	0
	SP	INF	INF	INF	INF	INF	INF	INF	INF	INF

*F*_15_	SR	**100**	**100**	**100**	**100**	0	90.00	**100**	**100**	**100**
	SP	4.93*E* + 04	5.63*E* + 04	5.63*E* + 04	5.63*E* + 04	INF	9.29*E* + 04	**9.08E + 03**	1.06*E* + 04	1.41*E* + 04

*F*_16_	SR	0	0	0	0	0	0	0	0	**100**
	SP	INF	INF	INF	INF	INF	INF	INF	INF	1.35*E* + 04

*F*_17_	SR	0	0	100	0	0	0	0	0	**100**
	SP	INF	INF	5.19*E* + 04	INF	INF	INF	INF	INF	**5.04E + 04**

*F*_18_	SR	0	0	100	0	0	0	0	0	**100**
	SP	INF	INF	**4.55E + 04**	INF	INF	INF	INF	INF	8.21*E* + 04

*F*_19_	SR	0	0	0	0	0	0	0	0	0
	SP	INF	INF	INF	INF	INF	INF	INF	INF	INF

*F*_20_	SR	0	0	0	0	0	0	0	0	0
	SP	INF	INF	INF	INF	INF	INF	INF	INF	INF

**Table 7:** Fm values achieved by PSO-W and PSO-DLP variants in 30-D problems.

	PSO-W	PSO-UEL	PSO-EEL	PSO-DLP
*F*_1_	1.34*E* − 117 ± 2.16*E* − 116	5.60*E* − 58 ± 6.46*E* − 59	5.93*E* − 323 ± **0.00E + 00**	**0.00E + 00** ± **0.00E + 00**

*F*_2_	1.41*E* − 45 ± 1.47*E* − 45	4.58*E* − 10 ± 6.29*E* − 10	6.94*E* − 184 ± 0.00*E* + 00	**3.00E − 86** ± **4.25E − 86**

*F*_3_	2.34*E* − 03 ± 9.83*E* − 04	1.20*E* − 03 ± 9.43*E* − 04	4.06*E* − 03 ± 1.90*E* − 03	**2.05E − 04** ± **1.24E − 05**

*F*_4_	2.53*E* + 01 ± 1.81*E* + 00	2.28*E* + 01 ± 6.61*E* − 01	4.78*E* − 17 ± 6.08*E* − 17	**2.01E − 19** ± **6.19E − 19**

*F*_5_	**7.11E − 15 ± 0.00E + 00**	2.84*E* − 14 ± 6.15*E* − 15	1.18*E* − 14 ± 4.10*E* − 15	**7.11E − 15 ± 0.00E + 00**

*F*_6_	7.39*E* − 03 ± 1.05*E* − 02	2.13*E* − 03 ± 2.09*E* − 03	6.68*E* − 02 ± 5.87*E* − 02	**0.00E + 00** ± **0.00E + 00**

*F*_7_	2.12*E* + 01 ± 1.02*E* + 01	**0.00E + 00** ± **0.00E + 00**	1.16*E* + 01 ± 1.52*E* + 00	**0.00E + 00** ± **0.00E + 00**

*F*_8_	2.37*E* + 01 ± 1.45*E* + 01	**0.00E + 00** ± **0.00E + 00**	3.33*E* − 01 ± 5.77*E* − 01	**0.00E + 00** ± **0.00E + 00**

*F*_9_	1.27*E* + 00 ± 4.43*E* − 01	7.53*E* − 01 ± 3.43*E* − 01	1.46*E* + 00 ± 5.58*E* − 01	**6.87E − 01** ± **3.60E − 01**

*F*_10_	2.51*E* + 01 ± 2.14*E* + 01	2.37*E* + 01 ± 2.50*E* − 01	5.70*E* − 01 ± 5.22*E* − 01	**2.00E − 01 ± 2.83E − 01**

*F*_11_	1.34*E* + 00 ± 3.14*E* − 01	3.20*E* − 14 ± 6.15*E* − 15	4.47*E* − 01 ± 7.74*E* − 01	**7.11E − 15 ± 0.00E + 00**

*F*_12_	3.93*E* − 02 ± 3.81*E* − 02	2.46*E* − 02 ± 3.64*E* − 02	1.40*E* − 02 ± 7.09*E* − 03	**3.70E − 03 ± 5.23E − 03**

*F*_13_	5.17*E* + 01 ± 2.25*E* + 01	4.21*E* + 01 ± 6.77*E* + 00	6.04*E* + 01 ± 9.56*E* + 00	**2.87E + 01** ± **3.68E + 00**

*F*_14_	4.05*E* + 01 ± 1.48*E* + 01	2.67*E* + 01 ± 3.06*E* + 00	5.40*E* + 01 ± **2.00E + 00**	**2.55E + 01** ± 1.34*E* + 01

*F*_15_	5.68*E* − 14 ± 0.00*E* + 00	1.14*E* − 13 ± 0.00*E* + 00	7.58*E* − 14 ± 3.28*E* − 14	**0.00E + 00** ± **0.00E + 00**

*F*_16_	1.04*E* + 01 ± 2.11*E* + 00	2.27*E* + 01 ± 5.57*E* − 01	**6.56E − 11 ± 6.74E − 11**	1.44*E* − 09 ± 3.21*E* − 09

*F*_17_	2.41*E* + 01 ± 1.19*E* + 01	1.89*E* − 13 ± 3.28*E* − 14	9.62*E* + 00 ± 5.11*E* + 00	**1.27E − 14** ± **1.48E − 15**

*F*_18_	2.51*E* + 01 ± 1.32*E* + 01	1.71*E* − 13 ± 5.68*E* − 14	5.11*E* + 00 ± 2.33*E* + 00	**4.55E − 14** ± **2.54E − 14**

*F*_19_	2.06*E* + 01 ± 6.74*E* − 01	**2.04E + 01** ± 1.85*E* − 01	2.05*E* + 01 ± **3.63E − 02**	**2.04E + 01 ± **6.25*E* − 02

*F*_20_	7.78*E* + 01 ± 1.38*E* + 01	5.93*E* + 01 ± 1.14*E* + 01	9.68*E* + 01 ± 4.34*E* + 01	**3.68E + 01 ± 2.81E + 00**

**Table 8:** Comparisons between PSO-DLP and other evolutionary algorithms in 30-D problems.

	CMA-ES	JADE	SADE	PSO-DLP
*F*_1_	4.83*E* − 131 ± 4.35*E* − 131	5.70*E* − 116 ± 3.19*E* − 116	4.57*E* − 81 ± 3.10*E* + 81	**0.00E + 00** ± **0.00E + 00**

*F*_2_	1.51*E* − 26 ± 3.61*E* − 27	2.84*E* − 75 ± 3.25*E* − 76	9.37*E* − 62 ± 5.44*E* − 63	**3.00E − 86** ± **4.25E − 86**

*F*_3_	5.31*E* − 01 ± 2.50*E* − 03	5.14*E* – 04 ± 2.14*E* − 05	1.12*E* − 03 ± 6.27*E* − 04	**2.05E − 04** ± **1.24E − 05**

*F*_4_	7.43*E* − 02 ± 3.64*E* − 03	7.09*E* − 02 ± 2.54*E* − 03	1.25*E* + 01 ± 3.18*E* + 00	**2.01E − 19** ± **6.19E − 19**

*F*_5_	7.11*E* − 15 ± 0.00*E* + 00	**4.45E − 15 ± 3.24E − 15**	1.18*E* − 14 ± 4.10*E* − 15	7.11*E* − 15 ± 0.00*E* + 00

*F*_6_	**0.00E + 00** ± **0.00E + 00**	**0.00E + 00** ± **0.00E + 00**	**0.00E + 00** ± **0.00E + 00**	**0.00E + 00** ± **0.00E + 00**

*F*_7_	1.54*E* + 01 ± 7.04*E* − 01	**0.00E + 00** ± **0.00E + 00**	**0.00E + 00** ± **0.00E + 00**	**0.00E + 00** ± **0.00E + 00**

*F*_8_	1.65*E* + 01 ± 4.95*E* + 00	**0.00E + 00** ± **0.00E + 00**	6.24*E* − 14 ± 3.54*E* − 15	**0.00E + 00** ± **0.00E + 00**

*F*_9_	**0.00E + 00** ± **0.00E + 00**	7.53*E* − 01 ± 3.43*E* − 01	1.46*E* + 00 ± 5.58*E* − 01	6.87*E* − 01 ± 3.60*E* − 01

*F*_10_	2.88*E* + 01 ± **2.20E − 02**	1.41*E* + 01 ± 4.31*E* + 00	2.98*E* + 01 ± 3.08*E* + 00	**2.00E − 01 ± **2.83*E* − 01

*F*_11_	1.03*E* + 00 ± 6.07*E* − 02	7.34*E* − 15 ± 1.56*E* − 15	**7.11E − 15 ± 0.00E + 00**	**7.11E − 15 ± 0.00E + 00**

*F*_12_	**0.00E + 00** ± **0.00E + 00**	1.23*E* − 02 ± 3.98*E* − 03	8.21*E* − 03 ± 9.21*E* − 04	3.70*E* − 03 ± 5.23*E* − 03

*F*_13_	3.12*E* + 01 ± 1.05*E* − 01	**2.81E + 01 ± 3.15E − 01**	3.14*E* + 01 ± 9.56*E* + 00	2.87*E* + 01 ± 3.68*E* + 00

*F*_14_	7.89*E* + 01 ± 3.58*E* − 01	2.71*E* + 01 ± 1.32*E* + 00	2.86*E* + 01 ± 3.45*E* − 01	**2.55E + 01** ± **1.34E + 01**

*F*_15_	1.77*E* − 26 ± 5.19*E* − 27	5.68*E* − 24 ± 3.42*E* − 24	4.78*E* − 23 ± 9.85*E* − 24	**0.00E + 00** ± **0.00E + 00**

*F*_16_	1.23*E* + 00 ± 2.50*E* − 03	1.02*E* + 01 ± 1.15*E* + 00	4.86*E* + 01 ± 5.78*E* + 00	**1.44E − 09 ± 3.21E − 09**

*F*_17_	1.94*E* + 01 ± 7.74*E* + 00	1.89*E* − 14 ± 3.28*E* − 15	1.62*E* − 13 ± 1.71*E* − 14	**1.27E − 14** ± **1.48E − 15**

*F*_18_	2.14*E* + 01 ± 6.34*E* + 00	1.71*E* − 13 ± 5.68*E* − 14	2.35*E* − 13 ± 5.24*E* − 14	**4.55E − 14** ± **2.54E − 14**

*F*_19_	2.12*E* + 01 ± **2.14E − 03**	2.09*E* + 01 ± 1.85*E* − 01	2.09 + 01 ± 4.61*E* − 02	**2.04E + 01 ± **6.25*E* − 02

*F*_20_	4.87*E* + 01 ± 1.12*E* + 01	3.95*E* + 01 ± 8.35*E* + 00*E*	5.67*E* + 01 ± 3.39*E* + 00	**3.68E + 01 ± 2.81E + 00**

Average rank	2.9	2.15	3.25	**1.2**

Final rank	3	2	4	**1**

## Appendix

Considering a case where *k* = 0.5, we calculate the joint probability distribution of *d*_*f*_ and *l*_*f*_:

(A.1) Fdf,lf=PDf≤df,Lf≤lf=Pkr1+1−kr2≤df,1−lfkr1−lf1−kr2≤0.

Since *r*_1_ and *r*_2_ are uniform random variables on [0,1], we can find

(A.2)

By the joint probability distribution of *d*_*f*_ and *l*_*f*_, the joint density of *d*_*f*_ and *l*_*f*_ is

(A.3) $$\begin{array}{c} f\left(\;d_{f},l_{f}\;\right)=\frac{F\left(d_{f},l_{f}\right)}{\partial d_{f}\partial l_{f}}=\left\{\begin{array}{cc} 4d_{f}, & 0\leq d_{f}\leq 1,\;\;\max\left(0,1-\frac{1}{2d_{f}}\right)\leq z\leq \min\left(1,\frac{1}{2d_{f}}\right)\\ 0, & \text{other.} \end{array}\right. \end{array}$$

Using the joint density of *d*_*f*_ and *l*_*f*_, we can calculate the marginal density of *l*_*f*_ and the marginal density of *d*_*f*_.

As

(A.4) $$\begin{array}{c} f_{L_{f}}\left(\;l_{f}\;\right)=\overset{+\infty }{\underset{-\infty }{\int }}f\left(\;d_{f},l_{f}\;\right)d\left(d_{f}\right), \end{array}$$

the marginal density of *l*_*f*_ is

(A.5) $$\begin{array}{c} f_{L_{f}}\left(\;l_{f}\;\right)=\left\{\begin{array}{cc} \frac{1}{\left(2{\left(1-l_{f}\right)}^{2}\right)}, & 0\leq l_{f}<0.5\text{<0.5}\\ \frac{1}{\left(2l_{f}^{2}\right)}, & 0.5\leq l_{f}\leq 1\text{<0.5}\\ 0, & \text{other.} \end{array}\right. \end{array}$$

The marginal density of *d*_*f*_ is

(A.6) $$\begin{array}{c} f_{D_{f}}\left(\;d_{f}\;\right)=\left\{\begin{array}{cc} 4d_{f}, & 0\leq d_{f}<\frac{1}{2}\\ 4\left(1-d_{f}\right), & \frac{1}{2}\leq d_{f}\leq 1\\ 0, & \text{otherwise.} \end{array}\right. \end{array}$$

We can get the conditional distribution of *d*_*f*_ given *l*_*f*_ using the following equation:

(A.7) $$\begin{array}{c} F_{D_{f}\;|\;L_{f}}\left(\;d_{f}\;|\;l_{f}\;\right)=\frac{\left(\partial /\partial l_{f}\right)F_{D_{f}L_{f}}\left(d_{f},l_{f}\right)}{\left(\partial /\partial l_{f}\right)F_{L_{f}}\left(l_{f}\right)}. \end{array}$$

So the conditional distribution of *d*_*f*_ given *l*_*f*_ is

(A.8) FDf ∣ Lfdf ∣ lf=4df21−lf2,0≤lf<12,  0≤df<121−lf1,0≤lf<12,  121−lf≤df≤14df2lf2,12≤lf<1,  0≤df<12lf1,12≤lf≤1,  12lf≤df≤1.

The conditional density of *d*_*f*_ given *l*_*f*_ is

(A.9) fDf ∣ Lfdf ∣ lf=8df1−lf2,0≤lf<12,  0≤df<121−lf8dflf2,12≤lf<1,  0≤df<12lf.
